# Multiplex PCR for detection of the *Vibrio* genus and five pathogenic *Vibrio* species with primer sets designed using comparative genomics

**DOI:** 10.1186/s12866-015-0577-3

**Published:** 2015-10-26

**Authors:** Hyun-Joong Kim, Ji-Oh Ryu, Shin-Young Lee, Ei-Seul Kim, Hae-Yeong Kim

**Affiliations:** Institute of Life Sciences & Resources and Graduate School of Biotechnology, Kyung Hee University, Yongin, 446-701 Republic of Korea

**Keywords:** Identification, PCR, Vibrios, *V. cholerae*, *V. parahaemolyticus*, *V. vulnificus*, *V. alginolyticus*, *V. mimicus*, Comparative genomics

## Abstract

**Background:**

The genus *Vibrio* is clinically significant and major pathogenic *Vibrio* species causing human *Vibrio* infections are *V. cholerae*, *V. parahaemolyticus*, *V. vulnificus*, *V. alginolyticus* and *V. mimicus*. In this study, we screened for novel genetic markers using comparative genomics and developed a *Vibrio* multiplex PCR for the reliable diagnosis of the *Vibrio* genus and the associated major pathogenic *Vibrio* species.

**Methods:**

A total of 30 *Vibrio* genome sequences were subjected to comparative genomics, and specific genes of the *Vibrio* genus and five major pathogenic *Vibrio* species were screened. The designed primer sets from the screened genes were evaluated by single PCR using DNAs from various *Vibrio* spp. and other non-*Vibrio* bacterial strains. A sextuplet multiplex PCR using six primer sets was developed to enable detection of the *Vibrio* genus and five pathogenic *Vibrio* species.

**Results:**

The designed primer sets from the screened genes yielded specific diagnostic results for target the *Vibrio* genus and *Vibrio* species. The specificity of the developed multiplex PCR was confirmed with various *Vibrio* and non-*Vibrio* strains. This *Vibrio* multiplex PCR was evaluated using 117 *Vibrio* strains isolated from the south seashore areas in Korea and *Vibrio* isolates were identified as *Vibrio* spp., *V. parahaemolyticus*, *V. vulnificus* and *V. alginolyticus*, demonstrating the specificity and discriminative ability of the assay towards *Vibrio* species.

**Conclusions:**

This novel multiplex PCR method could provide reliable and informative identification of the *Vibrio* genus and major pathogenic *Vibrio* species in the food safety industry and in early clinical treatment, thereby protecting humans against *Vibrio* infection.

**Electronic supplementary material:**

The online version of this article (doi:10.1186/s12866-015-0577-3) contains supplementary material, which is available to authorized users.

## Background

The *Vibrio* genus, which consists of more than 30 species, includes a number of major foodborne pathogens. Eleven of these *Vibrio* species are known to be human pathogens causing toxigenic cholera and other infections (vibriosis). *V. cholerae*, *V. parahaemolyticus*, *V. vulnificus*, *V. alginolyticus* and *V. mimicus* are pathogens of note in the clinical microbiology and food safety fields [1–6]. *Vibrio* are ubiquitous in halophilic marine environments and the consumption of raw or undercooked contaminated seafood causes human infections worldwide [4, 6–8]. The Cholera and Other *Vibrio* Illness Surveillance System (COVIS) of the Centers for Disease Control and Prevention (CDC) and the World Health Organization (WHO) considers pathogenic *Vibrio* species to be a public health threat and annually reports the number of human infections during *Vibrio* outbreaks (vibriosis by pathogenic *Vibrio* species including *V. parahaemolyticus*, *V. vulnificus*, *V. alginolyticus*, *V. mimicus* and other *Vibrio* species; Cholera due to toxigenic *V. cholerae*) [2, 9, 10].

Molecular biological DNA-based diagnostic methods, especially polymerase chain reaction (PCR), have been studied and developed for accurate and rapid identification of *Vibrio* spp. These methods provide advantages relative to and/or that complement standard microbiological culture-based methods [8, 11]. In early studies, PCR diagnostic methods were developed separately for each *Vibrio* species using specific expected virulence factor genes as genetic markers, including the cholera toxin (*ctx*) gene for *V. cholerae* and *V. mimicus* [8, 12], the thermostable direct hemolysin (*tdh*) gene and the thermostable direct hemolysin-related hemolysin (*trh*) gene for *V. parahaemolyticus* [13–15], and the cytotoxin-haemolysin (*vvhA*) gene for *V. vulnificus* [16, 17]. Recently, several genes, such as the regulatory gene *toxR* and the housekeeping genes *atpA*, *rpoB*, and *dnaJ*, have been suggested as novel genetic markers for use in PCR methods to complement the diagnosis of *Vibrio* species [3, 18–21]. Multiplex PCR methods for diagnosis of major pathogenic *Vibrio* species have also been developed [3, 21–25], and modified PCR methods with DNA-DNA hybridization or other technologies have been attempted for the accurate and rapid detection of *Vibrio* species [26–28].

In our previous studies, comparative genomics was used to screen for genetic markers for designing specific primer sets for *Salmonella* spp. and other pathogenic bacteria. The selected genetic markers were successfully used in the identification of *Salmonella enterica* serovars and other pathogenic bacteria, reflecting the potential application of genomics and bioinformatics for the detection and identification of foodborne pathogens [29–32]. Through the *Vibrio* genome projects, 21 *Vibrio* genomes have been completely sequenced and were available at the National Center for Biotechnology Information (NCBI) in 2012 (more than 100 draft *Vibrio* genome projects are also available or in progress). The original purpose of this study was to develop novel genetic markers, which would enable reliable and comprehensive diagnostics for *Vibrio* species. We employed an ‘*in silico*’ approach utilizing comparative genomics between genome sequences of *Vibrio* spp., thereby differentiating our method from previously reported *Vibrio* PCR methods. Specific expected genes for the *Vibrio* genus and 5 *Vibrio* species, which were chosen based on their significance as human pathogens as well as based on sequences being available through the NCBI, were screened using comparative genomics. Ultimately, a sextuplet *Vibrio* multiplex PCR was developed from the screened specific genes and the utility of this assay was evaluated.

## Results

### Screening of *Vibrio* genus- and species-specific genes using comparative genomics and the design of primer sets

To screen for *Vibrio* genus-specific genes, a total of 4832 gene sequences from *V. parahaemolyticus* RIMD 2210633 [GenBank: NC_004603.1, NC_004605.1] were compared with each representative genome sequence (fna file) from 4 *Vibrio* species. Ultimately, 1256 genes expected to be present in the *Vibrio* genus were selected based on the outputs of the Basic Local Alignment Search Tool (BLAST) program that indicated their relatively high matched DNA length (bp), thereby eliminating genes of low homology within the *Vibrio* genus. These genes were also compared to the non-redundant (nr) database of NCBI to eliminate genes that were highly matched with other biological sources. A total of 38 genes, which resulted low homology (less than 30 bp size that matched sequences in the nr database), were chosen and were compared individually to the nr and the microbial genome databases of the NCBI BLAST web site to again confirm their specificity in the *Vibrio* genus [33]. Finally, 2 genes (ATP synthase subunit alpha, C3281450-3279879 [GenBank: NC_004603.1] and recombinase A, C2694352-2693309 [GenBank: NC_004603.1]) were selected for the design and evaluation of primer sets for *Vibrio* genus-specific detection.

To screen for specific genes in each of the *Vibrio* species, the coding region sequences representing each of 5 *Vibrio* species were analyzed using the BLAST program to compare them against *Vibrio* genome sequences without the genome sequences of the targeted *Vibrio* species. The outputs of the BLAST program were analyzed by screening a number of genes (between 400 ~ 650 genes) to eliminate highly homologous genes within the *Vibrio* genus and to select those with matching under <100 (or 50) bp. The screened genes were then compared to the nr database of NCBI to select genes specific to the targeted *Vibrio* species with relatively low homology matching a length < 22 (or 23) bp of DNA. An appropriate number of specific expected genes (or DNA fragments) within the target *Vibrio* species were selected as follows: 59 genes from *V. parahaemolyticus* RIMD 2210633, 94 genes from *V. cholerae* O1 biovar El Tor str. N16961, 134 genes from *V. vulnificus* YJ016, 23 genes from *V. alginolyticus* 12G01 and 39 genes from *V. mimicus* MB-451. These selected genes were evaluated for their gene sizes and also were individually compared using the microbial genome database (complete and draft genome) on the NCBI BLAST web site [33]. In total, 15 primer sets (3 primer sets of each *Vibrio* species) expected to be specific for each species were designed. The designing of primers sets was considered by melting temperature (Tm), PCR product size and the regions of conserved (or varied) sequence in the target genes within *Vibrio* species.

### The specificity of designed primer sets for the *Vibrio* genus and species

The specificity of the designed primer sets was evaluated by conventional single PCR using various *Vibrio* species and other representative pathogenic bacteria, as listed in Table 1. In spite of the expected specificity of the genes screened through comparative genomics, 5 of 17 primer sets (one primer set from each of the five targeted *Vibrio* species) resulted in unexpected PCR results (i.e., amplification of non-targeted *Vibrio* species, unexpected PCR product size, or no PCR amplification), while the other 12 primer sets were shown to be specific, based on their targeting of the *Vibrio* genus and species (data not shown). For *Vibrio* genus-specific detection, two primer sets, C3281450 F36-R518 and C2694352 F46-R734, amplified specific 483-bp and 689-bp fragments with *Vibrio* strains and the primer set C2694352 F46-R734 was selected for multiplex PCR design. One primer set for each of the *Vibrio* species was selected based on their constant intensity of amplified PCR product and the lack of non-specific bands upon agarose gel analysis. For further confirmation of the PCR specificity of each primer set against the targeted *Vibrio* genus or species, the PCR products were sequenced and the sequence was compared with the sequence of the original target gene. All sequences were as expected (data not shown).

**Table 1:** Bacterial strains used in this study

Bacteria Strains	Source	Lane^a^	Bacteria Strains	Source	Lane
*Vibrio cholerae*	NCCP^b^ 13589	1	*Escherichia coli* EHEC (O157:H7)	ATCC 43890	23
*Vibrio alginolyticus*	ATCC^c^ 17749	2	*Escherichia coli* O157:H7	ATCC 43894	24
*Vibrio mimicus*	ATCC 33653	3	*Escherichia coli*	ATCC 35150	25
*Vibrio parahaemolyticus*	ATCC 27969	4	*Escherichia coli*	ATCC 11775	26
*Vibrio parahaemolyticus*	ATCC 33844	5	*Listeria monocytogenes*	ATCC 19113	27
*Vibrio parahaemolyticus*	ATCC 17802	6	*Listeria seeligeri*	ATCC 35967	28
*Vibrio vulnificus*	ATCC 33815	7	*Listeria innocua*	ATCC 33090	29
*Vibrio vulnificus*	ATCC 27562	8	*Salmonella enterica* serovar Typhimurium	ATCC 19585	30
*Vibrio vulnificus*	ATCC 33147	9	*Salmonella enterica* serovar Typhi	ATCC 33459	31
*Vibrio vulnificus*	ATCC 33814	10	*Salmonella enterica* serovar Enteritidis	ATCC 4931	32
*Vibrio aestuarianus*	ATCC 35048	11	*Salmonella enterica* serovar Gallinarum	ATCC 9184	33
*Vibrio algoinfesta*	KCCM^d^ 40861	12	*Salmonella enterica* serovar Pullorum	ATCC 9120	34
*Vibrio diazotrophicus*	ATCC 33466	13	*Shigella flexneri*	ATCC 12022	35
*Vibrio fluvialis*	ATCC 33809	14	*Shigella flexneri* 2a strain 2457 T	ATCC 700930	36
*Vibrio harveyi*	ATCC 35084	15	*Shigella dysenteriae*	ATCC 13313	37
*Vibrio harveyi*	ATCC 14126	16	*Shigella boydi*	ATCC 8700	38
*Vibrio mediterranei*	ATCC 43341	17	*Shigella boydi*	ATCC 9905	39
*Vibrio salmonicida*	ATCC 43839	18	*Shigella sonnei*	ATCC 25931	40
enteroinvensive *E. coli* (EIEC)	ATCC 43893	19	*Staphylococcus aureus*	ATCC 29737	41
enteroaggregative *E. coli* (EAEC)	NCCP 14039	20	*Stapylococcus epidermidis*	ATCC 14990	42
enteropathogenic *E. coli* (EPEC)	NCCP 14038	21	*Stapylococcus haemolyticus*	ATCC 29970	43
enterotoxigenic *E. coli* (ETEC)	NCCP 14037	22	*Yersinia enterocolitica*	ATCC 29913	44

^a^Lane, This number indicates the lane number in Fig. 1

^b^NCCP, National Culture Collection for Pathogens

^c^ATCC, American Type Culture Collection

^d^KCCM, Korean Culture Center of Microorganisms

### Specificity, sensitivity and multi-detection ability of *Vibrio* multiplex PCR

**Fig. 1:**
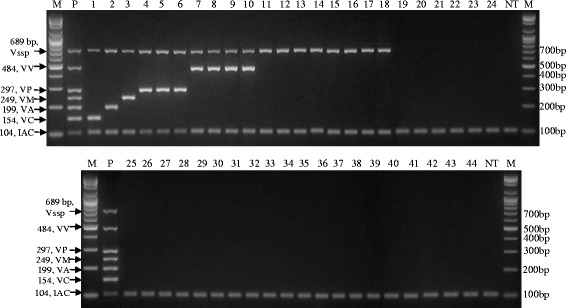
Results of the *Vibrio* multiplex PCR and its specificity using various genomic DNAs of *Vibrio* and other pathogenic bacterial type strains. M: 100-bp DNA ladder, P: Mixture of *Vibrio cholerae* KCDC 13589, *V. alginolyticus* ATCC 17749, *V. mimicus* ATCC 33653, *V. parahaemolyticus* ATCC 27969, *V. vulnificus* ATCC 33815, lanes 1 ~ lane 44: The information of type strains in each lane is indicated in Table 1, NT: no template. Vspp, *Vibrio* genus; VV, *V. vulnificus*; VP, *V. parahaemolyticus*; VM, *V. mimicus*; VA, *V. alginolyticus*; VC, *V. cholerae*

Based on the evaluation of each primer set by single conventional PCR, one of each of the specific primer sets for the *Vibrio* genus, *V. parahaemolyticus*, *V. cholerae*, *V. vulnificus*, *V. alginolyticus*, and *V. mimicus* was selected based on their Tm, the intensity of the amplified PCR product and PCR product size. A multiplex PCR reaction, including an internal amplification control (IAC, 104 bp), was constructed. Table 2 details their sources, primer concentrations, primer sequences and the expected PCR product sizes. The amount of the constructed plasmid used for the IAC and the concentration of each primer set were adjusted based on the amplified band intensities resulting from repeat multiplex PCR reactions using genomic DNAs from *Vibrio* strains. The discriminative ability and specificity of this *Vibrio* multiplex PCR was evaluated with various genomic DNAs of *Vibrio* and other non-*Vibrio* strains, as shown in Fig. 1. This multiplex PCR was designed to amplify 7 bands (lane P, Fig. 1) including the IAC, thereby enabling identification of the *Vibrio* genus and five major pathogenic *Vibrio* species, as shown in Fig. 1 (lanes 1 ~ 10: the specifically targeted *Vibrio* species; lanes 11 ~ 18: *Vibrio* spp. other than the specifically targeted *Vibrio* species). No false positive bands were amplified with other non-*Vibrio* bacterial strains using this multiplex PCR, which further demonstrates the specificity and discriminative ability of this *Vibrio* multiplex PCR. Additionally, the limit of detection (LOD) and evaluation using genomic DNA combinations from various *Vibrio* species were performed to confirm the sensitivity and multi-detection ability of the *Vibrio* multiplex PCR. Analysis of agarose gel electrophoresis loaded with 5 μl from a 25 μl-PCR showed the detection limit to be between 5 × 10^3^ and 5 × 10^2^ copies of *Vibrio* genomic DNA in a 25-cycle PCR and between 5 × 10^2^ and 5 × 10^1^ copies in a 30-cycle PCR, as shown in Additional file 1. The multi-detection ability was evaluated using a combination of genomic DNAs from *Vibrio* species and resulted in the specific amplification of fragments of the expected size and number with the different *Vibrio* species (Additional file 2).

**Table 2:** Primer pairs for *Vibrio* multiplex PCR designed and used in this study and their sources

Primer name	Source of gene^a^	Target genus or species	PCR product size (bp)	Final primer Conc. (μM)	Primer sequences (5’-3’)^b^	Protein of target gene
VP 1155272 F	NC_004605.1 (c1155272-1154856)	*Vibrio parahaemolyticus*	297	0.24	5’ AGCTT ATTGG CGGTT TCTGT CGG	hypothetical protein VPA1095
VP 1155272 R					5’ CKCAA GACCA AGAAA AGCCG TC	
VC C634002 F	NC_002506.1 (c634002-633547)	*Vibrio cholerae*	154	0.24	5’ CAAGC TCCGC ATGTC CAGAA GC	hypothetical protein VCA0694
VC C634002 R					5’ GGGGC GTGAC GCGAA TGATT	
VV 2055918 F79	NC_005139.1 (2055918–2056664)	*Vibrio vulnificus*	484	0.4	5’ CAGCC GGACG TCGTC CATTT TG	hypothetical protein VV2055
VV 2055918 R					5’ ATGAG TAAGC GTCCG ACGCG T	
VA 1198230 F	NZ_CH902589.1 (1198230–1198616)	*Vibrio alginolyticus*	199	0.1	5’ ACGGC ATTGG AAATT GCGAC TG	whole genome shotgun sequence
VA 1198230 R					5’ TACCC GTCTC ACGAG CCCAA G	
VM C727581F	NZ_ADAF01000001.1 (c727581-726859)	*Vibrio mimicus*	249	0.8	5’ ATAAA GCGGG CTTGC GTGCA	contig43, whole genome shotgun sequence
VM C727581R					5’ GATTT GGRAA AATCC KTCGT GC	
VG C2694352 F46	NC_004603.1 (c2694352-2693309)	*Vibrio* genus	689	1	5’ GTC ARA TTG AAA ARC ART TYG GTA AAG G	recombinase A
VG C2694352 R734					5’ ACY TTR ATR CGN GTT TCR TTR CC	

^a^Reference sequence number of chromosomes in GenBank at the NCBI and position of gene

^b^Mixed base: K = G + T; R = A + G; Y = C + T; N = A + C + G + T

### Evaluation of multiplex PCR with *Vibrio* isolates from seashore areas in Korea

**Fig. 2:**
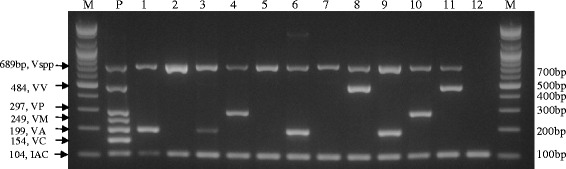
*Vibrio* multiplex PCR results with genomic DNAs of representative *Vibrio* isolates from the south seashore areas in Korea. M: 100-bp DNA ladder, P: Mixture of *Vibrio cholerae* KCDC 13589, *V. alginolyticus* ATCC 17749, *V. mimicus* ATCC 33653, *V. parahaemolyticus* ATCC 27969, *V. vulnificus* ATCC 33815, lane 1: Geoje-5, lane 2: Geoje −6, lane 3: Jinhae seawater-3, lane 4: Jinhae sand-water-3, lane 5: Jinhae sand-water-2, lane 6: Chungmu-1, lane 7: Chungmu-2, lane 8: MFDS Yeosu-3, lane 9: Busan-7, lane 10: Busan-6, lane 11: Busan-1, lane 12: no template. Vspp, *Vibrio* genus; VV, *V. vulnificus*; VP, *V. parahaemolyticus*; VM, *V. mimicus*; VA, *V. alginolyticus*; VC, *V. cholerae*

Isolated strains of *Vibrio* collected by the Ministry of Food and Drug Safety (MFDS), the Busan Metropolitan city Institute of Health & Environment (BMIHE), and our laboratory from the south seashore areas in Korea using microbiological culture-based procedures were used to evaluate the specificity/applicability of our multiplex PCR assay. A total of 117 isolates were subjected to *Vibrio* multiplex PCR and all of the *Vibrio* isolates showed positive results with the *Vibrio* genus-specific primer set (689 bp, VG C2694352 F46-R734), as shown in Table 3 and Fig. 2 (representative isolates are shown in Fig. 2). Among the 117 isolates, 94 strains resulted in a positive reaction, without any non-specific bands, with the *Vibrio* species-specific band identifying each as *V. parahaemolyticus*, *V. vulnificus* or *V. alginolyticus*, thereby reflecting the clear discriminative ability of the *Vibrio* multiplex PCR. No isolates of *V. cholerae* or *V. mimicus* were found in the environmental samples using this *Vibrio* multiplex PCR. Interestingly, 23 isolates were found to be *Vibrio* spp. other than the five targeted pathogenic *Vibrio* species, with a single amplification of the VG C2694352 F46-R734 primer set. Sequencing of the PCR products of the *Vibrio* isolates confirmed the expected amplification of each target gene, supporting the accuracy and specificity of the *Vibrio* multiplex PCR (data not shown). As a counterpart method to confirm the results of this *Vibrio* multiplex PCR with the *Vibrio* isolates, the representative *Vibrio* isolates in Fig. 2 were analyzed using Matrix-Assisted Laser-Desorption Ionization Time-of-Flight Mass Spectrometry (MALDI-TOF MS) and were identified same with the result of *Vibrio* multiplex PCR as shown in Table 4. [three strains of *Vibrio* species (lanes 2, 5 and 7) were identified as *Vibrio mytili* and *Vibrio fortis*; other nine strains were identified same *Vibrio* species as *Vibrio alginolyticus* (lanes 1, 3, 6 and 9) *Vibrio parahaemolyticus* (lanes 4 and 10) and *Vibrio vulnificus* (lanes 8 and 11)].

**Table 3:** Number of isolated *Vibrio* strains from local areas of Korea and their multiplex PCR results

Area of sampling	# of sample	Results of each specific primer set in multiplex PCR^a^					
		VP	VC	VV	VA	VM	*Vibrio* genus
		VP C1155272 F-R	VC C634002 F-R	VV 2055918 F79-R	VA 1198230 F-R	VM C727581 F-R	VG C2694352 F46-R734
Yeosu (by MFDS^b^)	20			20			20
Busan	8	2		5	1		8
Busan (by BMIHE^c^)	34	34					34
Geoje	14				4		14
Jinhae	34	5			21		34
Chungmu	7				2		7
Total	117	41	0	25	28	0	117

^a^VP, *V. parahaemolyticus*; VC, *V. cholerae*; VV, *V. vulnificus*; VA, *V. alginolyticus*; VM, *V. mimicus*

^b^MFDS, Ministry of Food and Drug Safety in South Korea

^c^BMIHE, Busan Metropolitan city Institute of Health & Environment

**Table 4:** Identification results of representative *Vibrio* isolates between *Vibrio* multiplex PCR and MALDI-TOF MS system

Identification of *Vibrio* isolates by *Vibrio* multiplex PCR		Identification of *Vibrio* isolates by MALDI-TOF MS analysis	
Lane No. in Fig. 2 ^a^	Description	MALDI-TOF MS results	log score value^b^
1	*Vibrio alginolyticus*	*Vibrio alginolyticus* DSM 2171 T	1.709
2	*Vibrio* spp.	*Vibrio mytili* DSM 19137 T	1.721
3	*Vibrio alginolyticus*	*Vibrio alginolyticus* DSM 2171 T	1.812
4	*Vibrio parahaemolyticus*	*Vibrio parahaemolyticus* DSM 11058	1.975
5	*Vibrio* spp.	*Vibrio mytili* DSM 19137 T	1.756
6	*Vibrio alginolyticus*	*Vibrio alginolyticus* DSM 2171 T	1.761
7	*Vibrio* spp.	*Vibrio fortis* DSM 19133 T	1.933
8	*Vibrio vulnificus*	*Vibrio vulnificus* DSM 10143 T	2.06
9	*Vibrio alginolyticus*	*Vibrio alginolyticus* CCM 2578 T	1.755
10	*Vibrio parahaemolyticus*	*Vibrio parahaemolyticus* DSM 11058	1.951
11	*Vibrio vulnificus*	*Vibrio vulnificus* CCUG 38429	2.144

^a^The number indicate lane number of *Vibrio* isolates in Fig. 2

^b^Isolates identification (log) score values, >2.0 indicated species-level, between 1.7 and 2.0 indicated genus level identification, respectively

## Discussion

The reliability of phenotype-based identification methods, which are laborious and time-consuming procedures that include microbiological culture-based identification, for detection of the *Vibrio* genus and specific *Vibrio* species has been questioned due to variations in the biochemical characteristics within the genus [4, 34]. In particular, the rapid differential diagnosis of clinically important *V. cholerae* from other *Vibrio* species is essential and economical and rapid diagnosis of *V. cholerae* is critical in mitigating the spread of *Vibrio* during an outbreak, as well as aiding in epidemic-preventing surveillance [12, 35–37]. Also, the current gold standard for laboratory diagnosis of cholera has been issued due to the lengthy culturing time required on selective growth media (TCBS is the only proven selective and ideal media for selective isolation and purification of *Vibrio* spp.) [4, 8, 12]. As an alternative to culture-based identification, molecular biological methods, especially PCR, have been developed for the detection and identification of pathogenic *Vibrio* species in the food and clinical microbiology fields. These methods aim to overcome the disadvantages of phenotype-based biochemical identification and to allow for reliable identification [8].

One of the impacts of our study on the development of a novel *Vibrio* multiplex PCR is that it provides an example of how to utilize comparative genomics with a limited genome database for practical application in diagnostics in food safety and clinical microbiology, particularly for the accurate identification of pathogenic bacteria. As shown in Table 5, our selected genetic marker genes were derived from comparative genomics using 30 genome sequences belonging to 19 *Vibrio* species, which are regarded as representative of these *Vibrio* species. We demonstrated the substantial potential of a limited bacterial genome database and bioinformatics technology in the diagnosis of human pathogens. In designing our *Vibrio* multiplex PCR, the selection of target *Vibrio* species was based on the reported number of human *Vibrio* infections and by the availability of genome sequences in the NCBI database, including *V. mimicus* and *V. alginolyticus*, which were ranked high among human infections caused by pathogenic *Vibrio* species [1, 4, 9, 38]. In addition, this *Vibrio* multiplex PCR method can be complemented or modified by the addition or subtraction of primer sets based on the availability of genome sequences or on the clinical significance of *Vibrio* species.

**Table 5:** Genome sequences of *Vibrio* strains used in this study^a^

Strain	Reference sequence^b^	Genome size (Mb)	Number of genes used in this study (on ffn file)	Release Date	Status of genome project
*V. parahaemolyticus* RIMD 2210633	NC_004603.1, NC_004605.1	5.17	4832	2003/03/05	Completed
*V. cholerae* O1 biovar EI Tor str. N16961	NC_002505.1, NC_002506.1	4.03	3834	2000/06/14	Completed
*V. cholerae* M66	NC_012578.1, NC_012580.1	3.94	3693	2009/04/20	Completed
*V. cholerae* MJ 1236	NC_012667.1, NC_012668.1	4.24	3772	2009/05/04	Completed
*V. cholerae* O395	NC_009456.1, NC_009457.1	4.13	3998	2007/05/08	Completed
*V. vulnificus* CMCP6	NC_004459.3, NC_004460.2	5.13	4433	2002/12/22	Completed
*V. vulnificus* MO6 24 O	NC_014966.1, NC_014965.1	5.01	4562	2011/01/24	Completed
*V. vulnificus* YJ016	NC_005140.1, NC_005139.1	5.26	5023	2003/10/01	Completed
*V. fischeri* MJ11	NC_011184.1, NC_011186.1	4.5	4039	2008/08/28	Completed
*V. fischeri* ES114	NC_006841.2, NC_006840.2	4.27	3817	2005/02/11	Completed
*V. anguillarum* 775	NC_015637.1, NC_015633.1	4.05	3732	2011/06/09	Completed
*V. species* Ex25	NC_013456.1, NC_013457.1	5.09	4518	2009/11/03	Completed
*V. harveyi* ATCC BAA-1116	NC_009783.1, NC_009784.1	6.06	6041	2007/08/28	Completed
*V. splendidus* LGP32	NC_011753.2, NC_011744.2	4.97	4432	2008/12/17	Completed
*V. alginolyticus* 12G01	NZ_CH902589 ~ NZ_CH902598^c^	5.16		2006/04/06	Scaffolds
*V. mimicus* MB-451	NZ_ADAF01000001 ~ NZ_ADAF01000003^C^	4.31		2009/10/23	Scaffolds

^a^The information in this table was updated in June 2012. Fourteen genome shotgun sequences of *Vibrio* strains are not listed in this table

^b^Completed genome sequences were obtained from genomes on the NCBI site (ftp://ftp.ncbi.nlm.nih.gov/genomes/) in December 2011

^c^The genome sequences of *V. alginolyticus* and *V. mimicus* were not completed. Scaffolds sequence data were obtained from the NCBI site and used for comparative genomics in this study

Our approach using an improved multiplex PCR assay along with comparative genomics for *Vibrio* identification differentiates our study from previously reported PCR methods. Recently, many *Vibrio* multiplex PCR assays have been reported for the identification of the major pathogenic *Vibrio* species; however, these multiplex PCR assays do not provide the diagnostic level required to be inclusive of the *Vibrio* genus [3, 21–26]. Our *Vibrio* multiplex PCR assay consists of two diagnostic levels for 1) the *Vibrio* genus and 2) five pathogenic *Vibrio* species. The reliable diagnosis of the *Vibrio* genus is important because the *Vibrio* genus consists of more than 30 species, including 11 reported human pathogenic *Vibrio* species [1, 4]. In addition to being able to diagnose the *Vibrio* genus, this multiplex PCR allows for the identification of the five major pathogenic *Vibrio* species: *V. cholerae*, *V. parahaemolyticus*, *V. vulnificus*, *V. alginolyticus* and *V. mimicus*. The ability to simultaneously identify the five major pathogenic *Vibrio* species in single reaction is valuable in the clinical and food microbiology fields in that it provides informative diagnostics at the *Vibrio* genus level as well as at the species level. Another distinct feature of our *Vibrio* multiplex PCR is the novel genetic markers for the five major pathogenic *Vibrio* species that were derived through the use of comparative genomics. These genetic markers are different from those in other previous reports in which marker gene selection was based on the functional qualities of the proteins encoded by their virulence-related/regulatory genes, or on phylogenetic classification of the housekeeping genes within *Vibrio* spp. [3, 8, 12–22]. We sought to acquire highly specific genetic marker genes for the diagnosis of *Vibrio* species by considering their presence/absence in the *Vibrio* genus and other closely related bacteria. We also considered the variable/conserved regions (sequence variation) in the genetic marker genes within *Vibrio* species. Furthermore, our screening approach for novel genetic markers was based purely on gene sequence comparisons using comparative genomics and was therefore not tied to the functions of genes and consequently the selected genetic markers were hypothetical proteins or proteins with other functions, as presented in Table 2. To give more objective validation on the presence of our screened genetic marker genes in each target *Vibrio* species (genus), each marker gene was compared and confirmed with each available *Vibrio* genome sequence of NCBI microbial genome database (Additional file 3). All each marker gene of *Vibrio* multiplex PCR was present in all available *Vibrio* genome sequences of NCBI microbial genome database (Complete Genome, Chromosome, Scaffold levels).

While screening marker genes of the *Vibrio* genus present in all *Vibrio* genome sequences (core genome of *Vibrio* genus), most of the *Vibrio* genes were eliminated. Ultimately, we were left with two genes specific to the *Vibrio* genus, despite the fact that they are present not only in *Vibrio* spp., but also in closely related bacteria: recombinase A, C2694352-2693309 [GenBank: NC_004603.1] and ATP synthase subunit alpha (*atpA*), C3281450-3279879 [GenBank: NC_004603.1] of *V. parahaemolyticus* RIMD 2210633. Interestingly, the *atpA* gene has already been reported in multiplex PCR assays for *Vibrio* species [3]. This supports the reliability of our genetic marker screening procedure using comparative genomics. Also, based on our screening results, we noted that the *atpA* gene is a more useful genetic marker for the *Vibrio* genus than for the *Vibrio* species.

The authors acknowledge that a more comprehensive panel of *Vibrio* strains will be required for the validation of this multiplex PCR. However, our study is extensive in that a large sample set of *Vibrio* isolates was sampled from seashore areas in Korea by MFDS, BMIHE and our laboratory. A total of 117 strains were evaluated by multiplex PCR and all isolates were determined to be *Vibrio* spp., as described in Table 3 and Fig. 2. Interestingly, while 94 isolates were identified as *V. parahaemolyticus*, *V. vulnificus* and *V. alginolyticus*, no isolates of *V. cholerae* or *V. mimicus,* which are considered to be more closely related to each other that to other *Vibrio* species [35, 37], were found. The results of the multiplex PCR assay that identified non-*V. cholerae* from seashore environmental samples are identical and comparable with those from a *Vibrio* monitoring study in live oysters by DePaola *et al*. [6], thereby supporting the specificity of our *Vibrio* multiplex PCR. Lastly, 23 isolates from among the 117 were identified as *Vibrio* spp., but were not among the 5 target *Vibrio* species used in this multiplex PCR, suggesting more informative diagnostics results with respect to other *Vibrio* species. Also, the identification of the representative *Vibrio* isolates using MALDI-TOF MS additionally supported the reliability of this *Vibrio* multiplex PCR as shown in Table 4.

## Conclusions

The present study selected novel genetic marker genes for the *Vibrio* genus and five other *Vibrio* species using comparative genomics and developed a sextuplet multiplex PCR assay using designed primer sets that allows for informative identification of *Vibrio*, thereby enabling rapid and specific diagnostics. We utilized this *Vibrio* multiplex PCR to demonstrate its discriminative ability for the *Vibrio* genus and each of five major pathogenic *Vibrio* species through the evaluation of *Vibrio* strains and isolates. However, despite the fact that additional validation will be needed with various *Vibrio* strains in order to establish the reliability of this *Vibrio* multiplex PCR, we suggest that our results with respect to the reliable performance of this assay should be of sufficient impact to recommend application of the assay as a useful diagnostic for pathogenic *Vibrio* species.

## Methods

### Bacterial strains

The *Vibrio* strains used in this study were collected from the American Type Culture Collection (ATCC), the Korean Culture Center of Microorganisms (KCCM), and the National Culture Collection for Pathogens (NCCP) of Korea as shown in Table 1. The *Vibrio* strains were inoculated in tryptic soy broth (BD, Sparks, MD, USA) containing 3 % NaCl and incubated using the recommended culture conditions for genomic DNA extraction. Various non-*Vibrio* type strains, including food-borne pathogens and other closely related bacterial type strains, were collected from the ATCC and NCCP, and incubated using the recommended culture conditions.

### Genome sequences of *Vibrio* species

Genome sequences and their *Vibrio* strain references used in this study are shown in Table 5 (14 uncompleted genome shotgun sequences of *Vibrio* strains are not shown in this table). A total of 14 completed genome sequences and 16 whole genome shotgun sequences (Scaffolds or contigs) of *Vibrio* strains, including *V. parahaemolyticus*, *V. cholerae*, *V. vulnificus*, *V. alginolyticus* and *V. mimicus,* were obtained from the National Center for Biotechnology Information (NCBI) web site [38] between December 2011 and April 2012.

### Comparative genomics for screening each *Vibrio* species-specific gene sequence

One representative genome sequence of each target *Vibrio* species was used for species-specific gene screening. These included: *Vibrio parahaemolyticus* RIMD 2210633 [GenBank: NC_004603.1, NC_004605.1] [36], *Vibrio cholerae* O1 biovar EI Tor str. N16961 [GenBank: NC_002505.1, NC_002506.1] [39], *Vibrio vulnificus* YJ016 [GenBank: NC_005139.ffn, NC_005140.ffn], *Vibrio alginolyticus* 12G01 [GenBank: NZ_CH902589 ~ NZ_CH902598] and *Vibrio mimicus* MB-451 [GenBank: NZ_ADAF01000001 ~ NZ_ADAF01000003] [37]. Scaffold sequences of *V. alginolyticus* and *V. Mimicus,* whose genome projects are not completed, were used for comparative genomics. To screen a specific gene (or DNA sequence) from each *Vibrio* species, the coding region sequences (ffn file) of each *Vibrio* species (target-*Vibrio* species) were compared against the genomic DNA sequences (fna file), which consist of *Vibrio* species excluding the genome sequence of the particular target *Vibrio* species, using the BLAST program (version 2.2.13) [40]. Based on BLAST analysis, we selected genes for each target *Vibrio* species that had low homology scores relative to the genomes of other *Vibrio* species and then re-compared them against the non-redundant (nr) DNA sequence NCBI database. Final candidate genes of each *Vibrio* species-specific were used for the design of primer sets.

### Comparative genomics for screening *Vibrio* genus-specific gene sequences

For screening *Vibrio* genus-specific genes, the coding region sequences of *Vibrio parahaemolyticus* RIMD 2210633 [GenBank: NC_004603.1, NC_004605.1] were used as the representative genome sequence of the *Vibrio* genus. The coding region sequences of *Vibrio parahaemolyticus* RIMD 2210633 (ffn file) were compared to each genome sequence of *V. cholerae*, *V. vulnificus*, *V. mimicus* and *V. alginolyticus* in order using the BLAST program and highly homologous genes expected to be present in all *Vibrio* species were screened. The screened genes were compared against the nr database and the microbial genomic database (representing complete and draft genome databases of microbes, respectively) on the NCBI web site. *Vibrio* genus-specific genes, which resulted in low homology (low sequence match) [33] considering their matched size and score of BLAST output, were selected for the design of *Vibrio* genus-specific primer sets.

### Genomic DNA extraction

Cultured media from each bacterial strain was harvested in microtubes and the genomic DNA of each strain was extracted using the Genomic DNA extraction kit for bacteria (iNtRON Biotechnology, Seoul, Korea), according to the manufacturers instructions. Genomic DNA concentration was measured using a UV-spectrophotometer (Model UV-1700, Shimadzu, Tokyo, Japan) and genomic DNAs with spectrophotometric ratios of 1.8 to 2.0 (A_260_/A_280_) were used. Genomic DNAs were stored at −20 °C.

### Primer construction and PCR conditions

Primer sets, expected to be specific to the *Vibrio* genus and/or species, were designed from each of the screened candidate genes and were evaluated using genomic DNAs of *Vibrio* and other type strains listed in Table 1. PCR amplifications were carried out with 200 μM of each dNTP, 0.5 unit of Ex *Taq* DNA polymerase (TaKaRa Bio Inc., Shiga, Japan), 1× Ex *Taq* buffer, 25 ng of template DNA and the adjusted concentration of each primer in a final reaction volume of 25 μl. PCR amplification was performed in a thermocycler (Model PC 808, ASTEC, Fukuoka, Japan) with an initial denaturation at 94 °C for 5 min, followed by 25 cycles of 94 °C for 30 s, 60 °C for 30 s, 72 °C for 30 s, finishing with a final extension at 72 °C for 10 min and storage at 4 °C thereafter. Amplified products were electrophoresed on a 3 % agarose gel in 0.5× Tris-acetate-EDTA buffer, stained with ethidium bromide, visualized under UV-irradiation and photographed with a digital camera (Model COOLPIX 4300, Nikon, Tokyo, Japan).

### Multiplex PCR of *Vibrio* and construction of the internal amplification control (IAC)

The multiplex PCR was designed to include six sets of screened primers, which targeted the *Vibrio* genus, *V. parahaemolyticus*, *V. cholerae*, *V. vulnificus*, *V. alginolyticus* and *V. mimicus*. The sequences of these primer sets along with their concentrations are shown in Table 2. In contrast to the single PCR reactions, one unit of Ex *Taq* DNA polymerase and 3 pg (around 10^6^ copies) of IAC template were used in a single multiplex PCR reaction. The IAC template was constructed using the sequence of the target gene, c1155272-1154856 [GenBank: NC_004605.1] in *Vibrio parahaemolyticus*. A primer set was designated as VP c1155272 IAC F (5’- AGCTTATTGGCGGTTTCTGTCGG CTACACCGTCGGCAGTGTGT -3’) and VP c1155272 IAC R (5’- CGCAAGACCAAGAAAAGCCGTC CTAGTGGCGTTTCGGAAAC -3’), which were flanked with the primer sequence of 1155272 F-R at the 5’ end, resulting in amplification of a 104-bp DNA fragment including the partial gene sequence of c1155272-1154856 of *V. parahaemolyticus*. The amplified DNA fragment was inserted into pGEM-T Easy Vector (Promega Corporation, Madison, WI) to generate the IAC template plasmid enabling the amplification of the 104 bp-PCR product with this internal control sequence by the 1155272 F-R primer set as a positive control for the *Vibrio* multiplex PCR.

### Limit of detection (LOD) and multi-detection ability for *Vibrio* species

For the LOD experiment using the *Vibrio* multiplex PCR, the quantity of *Vibrio* genomic DNAs was calculated as the copy number by genome size. As an example, for *Vibrio parahaemolyticus* (genome size of *Vibrio parahaemolyticus* RIMD 2210633: 5.17 Mb), 56.7 ng was considered to be 10^7^ copies of genomic DNA and was diluted from 10^6^ to 10^0^ copies per microliter. Diluted genomic DNA was added from 5 × 10^6^ copies to 5 × 10^0^ copies in each reaction and 5 μl of the 25 μl-PCR products was loaded for 3 % agarose gel electrophoresis. The multi-detection ability of the *Vibrio* multiplex PCR was also evaluated with various combinations of genomic DNAs from the five *Vibrio* species (1 ng per each *Vibrio* species sample).

### Collection and isolation of *Vibrio* isolates from the south seashore areas in Korea and evaluation of multiplex PCR

The isolated strains of *V. parahaemolyticus and V. vulnificus* sampled from the seashore of Busan and Yeosu in South Korea were obtained from BMIHE and MFDS in South Korea, respectively. Other *Vibrio* strains were isolated from 4 south seashore areas, Busan, Geoje, Jinhae, and Chungmu, in Korea, using isolation methods recommended by the bacteriological analytical manual from the FDA [8]. In brief, samples from each local area were kept at 7 to 10 °C until delivered to the laboratory. Then, 25 g (or 50 ml of liquid) of sample was placed into a stomacher bag and 225 ml of phosphate buffered saline (PBS) was added. Samples were homogenized for 1 min at maximum RPM using a stomacher (Seward Stomachers® 400 Circulator, Manchester, UK). One milliliter of homogenized sample was inoculated into 10 ml of alkaline peptone water (APW) and was incubated overnight at 35 °C. An inoculating loop was used to streak bacteria from the top of the APW onto thiosulfate-citrate-bile salt-sucrose Agar (TCBS agar, BD, Sparks, MD, USA) and the plate was incubated overnight at 35 °C. *Vibrio* positive colonies, which were yellow or green to bluish-green colonies on TCBS agar, were sampled and cultured in TSB media containing 3 % NaCl for isolation of stock or genomic DNA extraction allowing for use in multiplex PCR.

### PCR product sequencing

Each amplified PCR product was purified from agarose gels using the QIAquick Gel Extraction Kit (Qiagen GmbH, Hilden, Germany) and by QIAquick PCR Purification Kit (Qiagen). The sequencing of purified PCR products was performed using an automated DNA sequencer (Applied Biosystems, Foster City, CA, USA) using the forward and reverse primers used in the *Vibrio* multiplex PCR. The sequencing data was compared with the known targeted gene sequences which were originally used for specific-primer design for each *Vibrio* species.

### MALDI-TOF MS analysis

For the identification of *Vibrio* isolates by means of MALDI-TOF MS, an individual colony were deposited directly on a target polished steel microscout target plate (MSP 96; Bruker Daltonik GmbH, Bremen, Germany) overlaid with 1 μl of 70 % formic acid and 1 μl of α-cyano-4-hydroxycinnamic acid matrix solution in acetonitrile : water : trifluoro acetic acid (TFA) (ratio 50:47.5:2.5, v/v) and then air-dried. After crystallization, measurements were performed on a microflex LT bench-top mass spectrometer (Bruker Daltonik GmbH) with a smart beam laser. The parameter conditions were as follows: ion source 1, 20.0 kV; ion source 2, 18.2 kV; lens, 6.0 kV; initial laser power; 25 %; maximal laser power; 35 %. Ionization was performed with laser irradiation. Raw spectra data were imported into Biotyper software 3.0 (Bruker Daltonik GmbH). Mass spectra were collected within a mass range of 2000–20,000 m/z, with 1200 satisfactory laser shots in 240 shot steps. Prior to analysis, the reference strain *Escherichia coli* DH5α was used as a standard for calibration and as reference for quality control. Each sample was matched to a reference library in the Biotyper software database, which contains spectra of approximately 5627 species.

## Additional files

Additional file 1:
**(Figure) Limit of detection (LOD) results of** ***Vibrio*** **multiplex PCR.** Panel (A) 25 cycles, Panel (B) 30 cycles, loaded amount of PCR product was 5 μl from 25 μl PCR product. M: 100 bp ladder, 1: 5 × 10^6^ copies, 2: 5 × 10^5^ copies, 3: 5 × 10^4^ copies, 4: 5 × 10^3^ copies, 5: 5 × 10^2^ copies, 6: 5 × 10^1^ copies, 7: 5 × 10^0^ copies, 8: No Template (NT); Vspp, *Vibrio* genus; VC, *Vibrio cholerae* KCDC 13589; VA, *V. alginolyticus* ATCC 17749; VM, *V. mimicus* ATCC 33653; VP, *V. parahaemolyticus* ATCC 27969; VV, *V. Vulnificus* ATCC 33815. (PPTX 321 kb) Additional file 2:
**(Figure) Results of** ***Vibrio*** **multiplex PCR with genomic DNA combinations from** ***Vibrio*** **species.** M: 100-bp ladder, 1: VC VA, 2: VC VM, 3: VC VP, 4: VC VV, 5: VA VM, 6: VA VP, 7: VA VV, 8: VM VP, 9: VM VV, 10: VP VV, 11: VC VA VM, 12: VC VM VP, 13: VC VM VV, 14: VC VA VV, 15: VA VM VP, 16: VA VM VV, 17: VA VP VV, 18: VM VP VV, 19: VC VA VM VP, 20: VC VM VP VV, 21: VA VM VP VV, 22: VC VA VP VV, 23: VC VA VM VP VV, 24: NT. Vspp, *Vibrio* genus; VC, *Vibrio cholerae* KCDC 13589; VA, *V. alginolyticus* ATCC 17749; VM, *V. mimicus* ATCC 33653; VP, *V. parahaemolyticus* ATCC 27969; VV, *V. Vulnificus* ATCC 33815. (PPTX 382 kb) Additional file 3:
**(Tables) Presences of the six marker genes used for the** ***Vibrio*** **multiplex PCR in each genome of NCBI microbial genome database.** The lists of genomes (Column A) were obtained from NCBI genome database (http://www.ncbi.nlm.nih.gov/genome/browse/). The matched genome sequence (Column C), ID and position of sequence (Column D) are BLAST results at microbial genome BLAST web site in NCBI (http://blast.ncbi.nlm.nih.gov/Blast.cgi?PAGE_TYPE=BlastSearch&BLAST_SPEC=MicrobialGenomes). (XLSX 78 kb)
