# Supplementary material for: Multiplex PCR for detection of the Vibrio genus and five pathogenic Vibrio species with primer sets designed using comparative genomics
Source: BMC Microbiol. 2015 Oct 26;15:239. doi: 10.1186/s12866-015-0577-3 (PMC4624192; doi:10.1186/s12866-015-0577-3)
Supplement: Additional file 1: — (Figure) Limit of detection (LOD) results of Vibrio multiplex PCR. Panel (A) 25 cycles, Panel (B) 30 cycles, loaded amount of PCR product was 5 μl from 25 μl PCR product. M: 100 bp ladder, 1: 5 × 10^6^ copies, 2: 5 × 10^5^ copies, 3: 5 × 10^4^ copies, 4: 5 × 10^3^ copies, 5: 5 × 10^2^ copies, 6: 5 × 101 copies, 7: 5 × 100 copies, 8: No Template (NT); Vspp, Vibrio genus; VC, Vibrio cholerae KCDC 13589; VA, V. alginolyticus ATCC 17749; VM, V. mimicus ATCC 33653; VP, V. parahaemolyticus ATCC 27969; VV, V. Vulnificus ATCC 33815. (PPTX 321 kb) [file 12866_2015_577_MOESM1_ESM.pptx]

## Slide 1
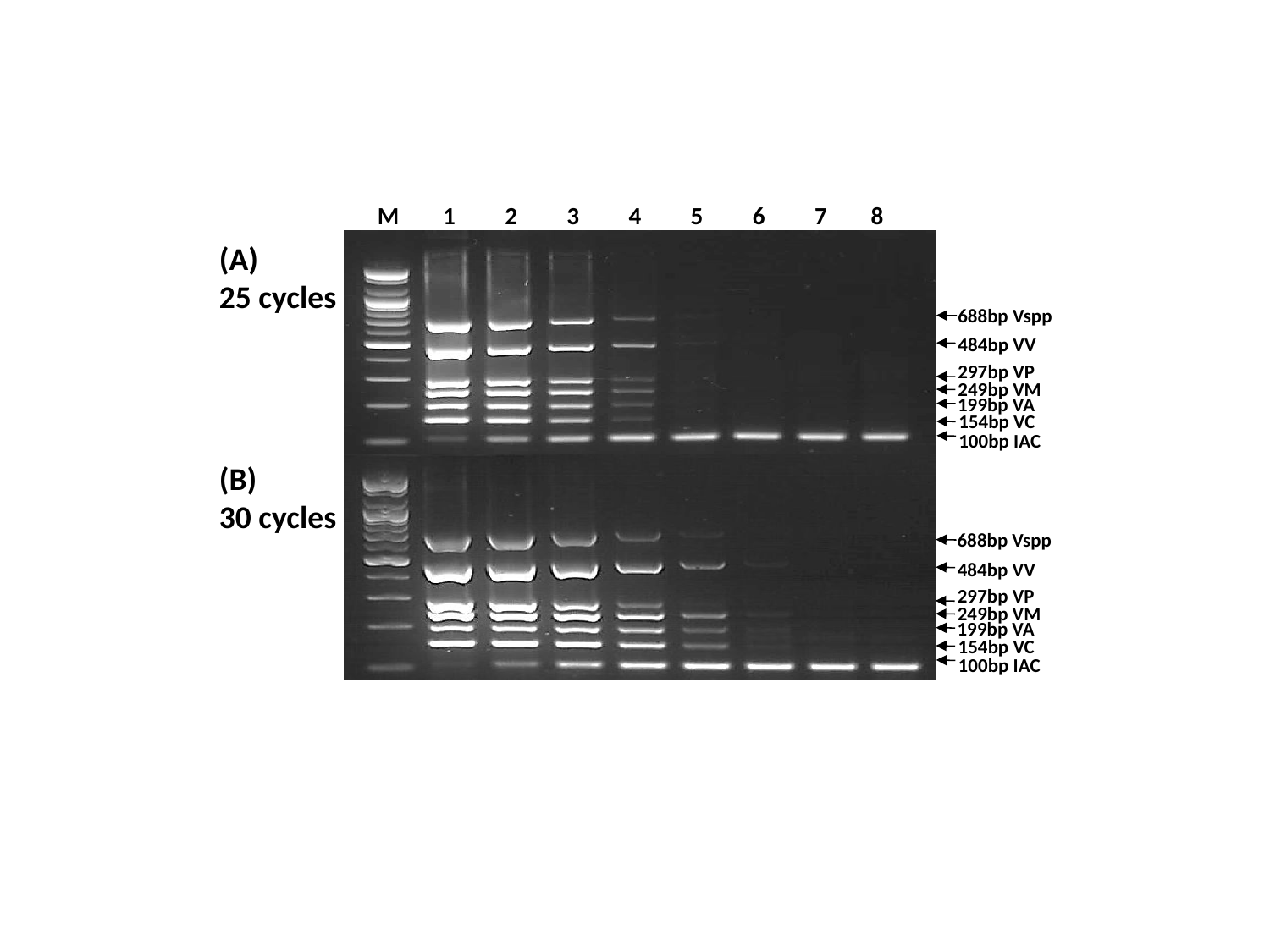

M 1 2 3 4 5 6 7 8
(A)
25 cycles
688bp Vspp
484bp VV
297bp VP
249bp VM
199bp VA
154bp VC
100bp IAC
(B)
30 cycles
688bp Vspp
484bp VV
297bp VP
249bp VM
199bp VA
154bp VC
100bp IAC
