# Supplementary figures and images for: Multiplex PCR for detection of the Vibrio genus and five pathogenic Vibrio species with primer sets designed using comparative genomics
Source: BMC Microbiol. 2015 Oct 26;15:239. doi: 10.1186/s12866-015-0577-3 (PMC4624192; doi:10.1186/s12866-015-0577-3)

## Slide 1
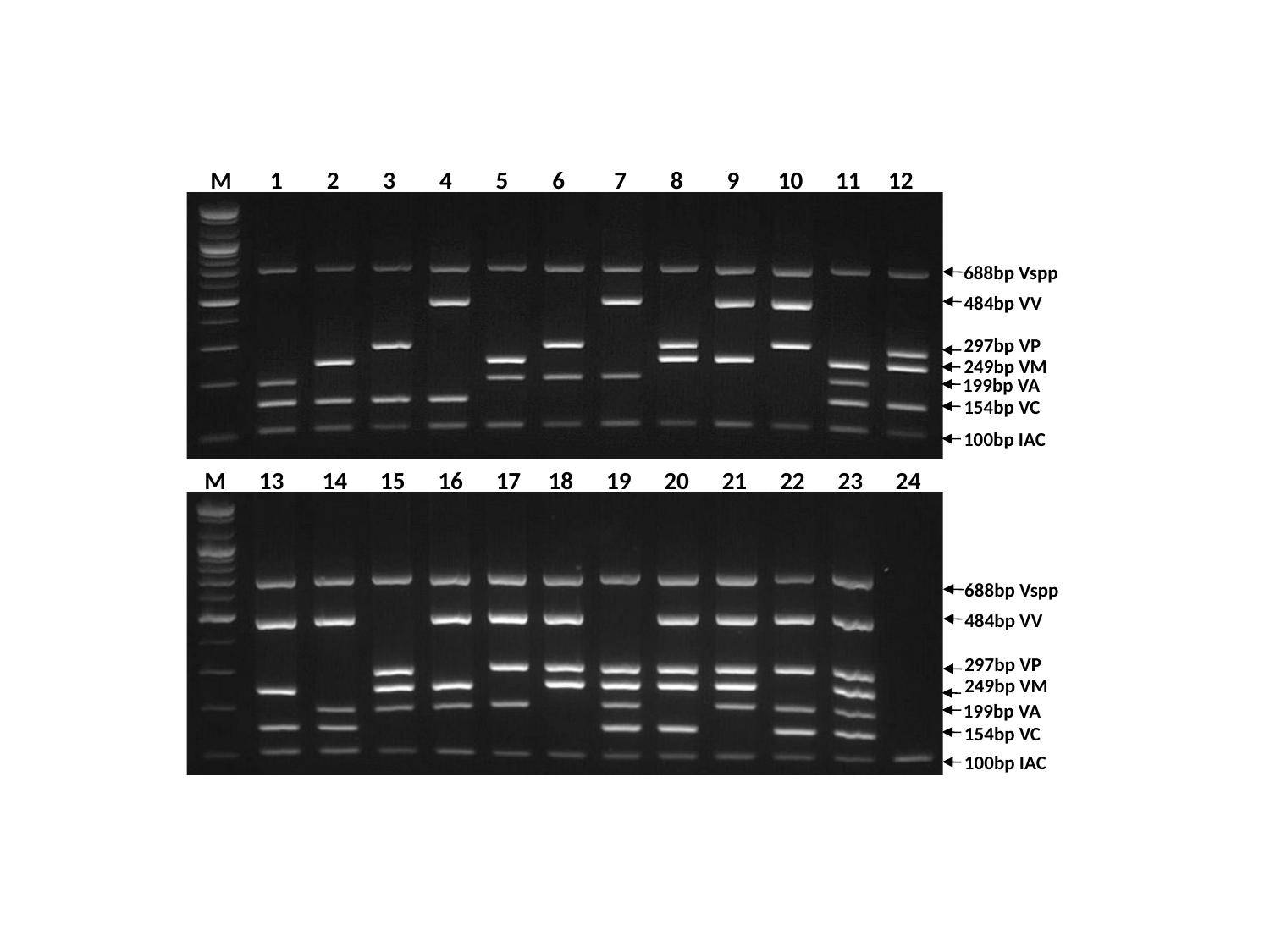

M 1 2 3 4 5 6 7 8 9 10 11 12
688bp Vspp
484bp VV
297bp VP
249bp VM
199bp VA
154bp VC
100bp IAC
M 13 14 15 16 17 18 19 20 21 22 23 24
688bp Vspp
484bp VV
297bp VP
249bp VM
199bp VA
154bp VC
100bp IAC

Supplement: Additional file 2: — (Figure) Results of Vibrio multiplex PCR with genomic DNA combinations from Vibrio species. M: 100-bp ladder, 1: VC VA, 2: VC VM, 3: VC VP, 4: VC VV, 5: VA VM, 6: VA VP, 7: VA VV, 8: VM VP, 9: VM VV, 10: VP VV, 11: VC VA VM, 12: VC VM VP, 13: VC VM VV, 14: VC VA VV, 15: VA VM VP, 16: VA VM VV, 17: VA VP VV, 18: VM VP VV, 19: VC VA VM VP, 20: VC VM VP VV, 21: VA VM VP VV, 22: VC VA VP VV, 23: VC VA VM VP VV, 24: NT. Vspp, Vibrio genus; VC, Vibrio cholerae KCDC 13589; VA, V. alginolyticus ATCC 17749; VM, V. mimicus ATCC 33653; VP, V. parahaemolyticus ATCC 27969; VV, V. Vulnificus ATCC 33815. (PPTX 382 kb) [file 12866_2015_577_MOESM2_ESM.pptx]
